# Hybrid Autofluorescence and Optoacoustic Microscopy for the Label-Free, Early and Rapid Detection of Pathogenic Infections in Vegetative Tissues

**DOI:** 10.3390/jimaging9090176

**Published:** 2023-08-29

**Authors:** George J. Tserevelakis, Andreas Theocharis, Stavroula Spyropoulou, Emmanouil Trantas, Dimitrios Goumas, Filippos Ververidis, Giannis Zacharakis

**Affiliations:** 1 Foundation for Research and Technology Hellas, Institute of Electronic Structure and Laser, N. Plastira 100, GR-70013 Heraklion, Crete, Greece; tserevel@iesl.forth.gr (G.J.T.); ph5090@edu.physics.uoc.gr (S.S.); 2 Department of Agriculture, School of Agricultural Sciences, Hellenic Mediterranean University, Estavromenos, GR-71410 Heraklion, Crete, Greece; theoxarisandr@hotmail.com (A.T.); mtrantas@hmu.gr (E.T.); dgoumas@hmu.gr (D.G.); 3 Institute of Agri-Food and Life Sciences, University Research Centre, Hellenic Mediterranean University, GR-71410 Heraklion, Crete, Greece

**Keywords:** optoacoustic, photoacoustic, fluorescence, microscopy, rapid disease diagnosis, pathogen detection, *Xanthomonas campestris*, *Xylella fastidiosa*

## Abstract

Agriculture plays a pivotal role in food security and food security is challenged by pests and pathogens. Due to these challenges, the yields and quality of agricultural production are reduced and, in response, restrictions in the trade of plant products are applied. Governments have collaborated to establish robust phytosanitary measures, promote disease surveillance, and invest in research and development to mitigate the impact on food security. Classic as well as modernized tools for disease diagnosis and pathogen surveillance do exist, but most of these are time-consuming, laborious, or are less sensitive. To that end, we propose the innovative application of a hybrid imaging approach through the combination of confocal fluorescence and optoacoustic imaging microscopy. This has allowed us to non-destructively detect the physiological changes that occur in plant tissues as a result of a pathogen-induced interaction well before visual symptoms occur. When broccoli leaves were artificially infected with *Xanthomonas campestris* pv. *campestris* (*Xcc*), eventually causing an economically important bacterial disease, the induced optical absorption alterations could be detected at very early stages of infection. Therefore, this innovative microscopy approach was positively utilized to detect the disease caused by a plant pathogen, showing that it can also be employed to detect quarantine pathogens such as *Xylella fastidiosa*.

## 1. Introduction

The increasing population and subsequent food consumption is a major challenge that the world must overcome to limit issues with food accessibility and malnutrition, both of which are crucial parameters of food security [1,2]. Agriculture plays a pivotal role to this end, as it is responsible for the production of the primary sources of both food for humans and for the animal feed that supports the production of meat, another primary source of food for humans. To meet increasing demands by 2050, global agricultural production may need to be substantially increased to follow the increases in the human population, meat and dairy consumption, and in biofuel production [3].

Although an increase in agricultural yields is expected, this increase will not be sufficient to double global crop production by 2050 [4]; pests and pathogens reduce the yield and quality of agricultural production and restrict trade and consumption, complicating efforts to achieve food security [5]. Moreover, there is a risk of the introduction of new diseases, as endogenous plants have no natural defenses and therefore are highly susceptible [6]. This indicates that, in order to enhance the performance of global agriculture and associated food systems, containment and control of crop pests and pathogens must be considered. Particularly in the case of quarantine pathogens, governments and international organizations have collaborated to establish robust phytosanitary measures, promote disease surveillance, invest in research and development, and educate farmers on disease management through good agricultural practices to mitigate the impact on food security.

Classic as well as modernized tools for disease and pathogen surveillance and diagnosis exist [7], but most of these are time consuming, laborious, or are less sensitive. Traditional methods that are based on the identification of a plant disease or pest attack through phenotypical symptoms are simpler to perform but sample preparation and analysis are laborious and fail to produce quantitative results. On the other hand, existing modern tools are quicker and may produce more reliable results in terms of sensitivity and specificity [8]. Exceptional methods currently involved in disease detection, are the enzyme-linked immunosorbent assay (ELISA) to detect pathogen proteins [9], the regular or quantitative real-time polymerase chain reaction (RT-PCR or qPCR) [10,11], loop-mediated isothermal amplification (LAMP) to detect pathogen nucleic acids [12], sequencing, microarray analysis [13], specific biosensors [14], volatile organic compound analysis for pathogen detection [15,16], and special spectroscopic properties [17]. The latest additions to the plant pathology quiver are droplet digital PCR [18] and methods based on next-generation sequencing (NGS) approaches [19]; however, although promising, these rely heavily on an expensive array of instrumentation for genetic as well as for bioinformatic analyses.

Beyond sensitivity, rapid detection is among the most critical features of diagnostic tests, especially those intended for quarantine pests (European and Mediterranean Plant Protection Organization A1 and A2 lists [20] or equivalent lists of another world region) as special countermeasures should be taken as fast as possible to limit the spread of the disease. A certain limitation of the current state-of-the-art methodologies derives from the current absence of a universal detection method that is able to detect pathogens before the expression of a disease’s phenotypic visual symptoms. Such a system will be able to assist a country’s border customs control from the importation of infected plant material, particularly those imported for propagation purposes. The latter is of special importance as, when plants or plant products arrive at a country’s border, they may be held in quarantine for a length of time, especially when plant material requires a period of growth to ensure the absence of pests and diseases [21]. In this work, we demonstrate an instrumental prototype layout for the direct non-genetic and immediate detection of microbial pathogens inside the tissues of infected plants, well before visual disease symptoms can even appear. This custom-developed hybrid system integrates state-of the-art confocal fluorescence and optoacoustic microscopy modalities to provide complementary label-free imaging contrast at high spatial resolution. While such multimodal instruments have been previously employed for several imaging applications, including the sensitive detection of pigments in plant leaves [22], the examination of various ocular tissues [23,24], the investigation of emerging models organisms [25], as well as the quantification of melanin in fish [26], this is the first time to our knowledge that they have been utilized for the early and rapid diagnosis of pathogenic infections in plants, paving the way for new and significant developments in the field.

## 2. Materials and Methods

### 2.1. Hybrid Microscopy System

**Figure 1:**
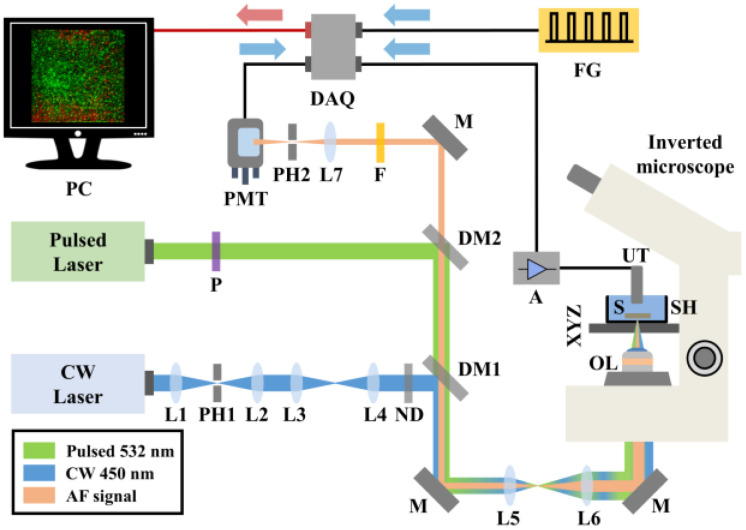
Schematic representation of the hybrid confocal fluorescence and optoacoustic microscopy system for the investigation of vegetative tissues. L (1–7), lenses; M, mirror; PH (1–2), pinholes; ND, neutral density filters; DM (1–2), dichroic mirrors; F, optical filter; PMT, photomultiplier tube; AF, autofluorescence; CW, continuous wave; P, linear polarizer; OL, objective lens; XYZ, 3D translation stages; SH, sample holder; S, specimen; UT, ultrasonic transducer; A, RF amplifier; DAQ, data acquisition card; FG, function generator; PC, recording computer.

A detailed technical description of the hybrid confocal fluorescence and optoacoustic microscopy setup (Figure 1) has been provided in a previous study [26]. In brief, autofluorescence (AF) excitation is achieved by employing a low-power continuous wave (CW) diode laser at 450 nm (CPS450, Thorlabs, Newton, NJ, USA; output power 4.5 mW). The emitted optical radiation is spatially filtered using a telescopic optical system comprising two positive lenses (L1, L2) and a pinhole (PH1), thus improving the beam quality. A second optical system consisting of two additional positive lenses (L3, L4) is used to precisely control the divergence of the beam and achieve a confocal configuration with the optoacoustic excitation laser source across the sample plane. A set of neutral density (ND) filters sufficiently attenuates the optical radiation, avoiding potential photodamage effects on the investigated vegetative tissue. Subsequently, a long-pass dichroic mirror (DM1; DMLP505, Thorlabs, Newton, NJ, USA; cut-off wavelength: 505 nm) reflects the excitation light into a beam expanding telescope (L5, L6), whereas a broadband mirror (M) placed at 45 degrees is used to guide the laser beam into a modified inverted microscope serving as the platform of the hybrid imaging system. An objective lens (OL; Achromat 8X, LOMO, St. Petersburg, Russia) of effective numerical aperture equal to 0.1 focuses the light on the examined specimen (S), which in turn is fixed at the bottom of an optically transparent sample holder (SH) by applying a thin ultrasound gel layer. The SH is attached to a three-dimensional translational motion system (XYZ), which is used for the specimen’s initial positioning and raster-scanning over the focused beams (8MTF-75LS05, Standa, Vilinius, Lithuania), to obtain an image through point-by-point data acquisition. The back-scattered AF light is collected by the OL, transmitted through the long-pass dichroic mirrors DM1 and DM2 (DMLP550, Thorlabs, Newton, NJ, USA; cut-off wavelength: 550 nm) and finally filtered by a long-pass filter (F; FGL570, Thorlabs, Newton, NJ, USA; cut-off wavelength: 570 nm), cutting off any reflected excitation photons. A converging lens (L7) focuses AF light through a pinhole (PH2) to reject out of focus signals, in a confocal configuration with the OL. The transmitted in-focus AF is detected by a photomultiplier tube (PMT; H6780-20, Hamamatsu, Hamamatsu City, Japan) of high sensitivity, whereas the respective signals are digitized by a data acquisition card (DAQ; PCIe-9852, ADLINK, Taipei, Taiwan; sampling rate: 200 MS/s; bandwidth: 90 MHz) and recorded by a computer (PC).

Regarding the optoacoustic imaging path, a variable repetition rate Q-switched nanosecond laser emitting at 532 nm (QIR-1064-200-S, CrystaLaser LC, Reno, NV, USA; maximum pulse energy: 29.4 µJ, pulse duration: 10 ns, frequency-doubled through second harmonic generation) is utilized for the efficient excitation of the signals. The laser source is externally triggered by an arbitrary function generator (FG; 33600A Series Trueform, Keysight Technologies, Santa Rosa, CA, USA) at a selected pulse repetition rate of 10 kHz, synchronizing the data acquisition and raster scanning procedures. A linear polarizer (P) is used to reduce the optical power of the pulsed beam prior to its reflection by DM2 and subsequent transmission through DM1, resulting in a spatial overlap with the AF excitation radiation. Similarly, the pulsed light passes through the beam expander (L5, L6) and is guided into the modified microscope, where it is focused by the OL on the sample. The emitted ultrasonic optoacoustic waves are detected by a spherically focused piezoelectric ultrasonic transducer (UT; V373-SU, Olympus, Tokyo, Japan; − 6 dB bandwidth: 13.3–32.9 MHz, Focal distance: 31.3 mm) which is immersed in the SH filled with distilled water. The distilled water serves as a coupling medium between the optoacoustic sources and the UT, enabling the effective transmission and detection of the generated ultrasonic waves. The optoacoustic signals are amplified by two low-noise RF amplifiers (A; TB-414-8A+, Mini-Circuits, Camberley, England) prior to their digitization and recording by the same DAQ card, concurrently with the AF channel. To enhance the signal-to-noise ratio (SNR), 20 waveforms are averaged for the acquisition of each pixel value comprising the maximum amplitude projection (MAP) optoacoustic image. The total time required for the recording of a typical 500-by-500 pixel hybrid image is approximately 20 min. Pulse energy on the sample’s focal plane is measured at 120 nJ, whereas the respective optical power of the CW laser is equal to 400 μW. The lateral resolution of the system is ultimately determined by the optical diffraction limit, and is estimated to be ~2.5 μm, for both of the integrated imaging modalities. Control and synchronization of the hybrid microscope is accomplished by means of custom-developed software. Finally, all data are processed using MATLAB 2013programming environment and ImageJ 1.52a image processing software.

### 2.2. Inoculum Preparation, Plant Development and Artificial Inoculations

For the implementation of artificial inoculations, the *Xanthomonas campestris* pv. *campestris* strain 8004 (*Xcc*), registered to the Hellenic Mediterranean University microbial culture repository, was used. The bacteria were maintained at −80 °C in 20% (*v*/*v*) of glycerol until use. A fresh culture was prepared by inoculating a single colony into 5 mL of LB medium (1% tryptone, 0.5% yeast extract, 1% NaCl) and left to grow at 27 °C with shaking at 230 rpm. After 12 h, the OD_600_ of the culture was adjusted to 0.1 with sterile water and used as inoculum. 

Seeds of broccoli *Brassica oleracea* var. *italica* were surface sterilized by rinsing them in 70% ethanol for 1–2 min followed by a 15 min exposure to 1.3% sodium hypochlorite. The seeds were left to germinate in the dark and, after three days, were transferred in pots with soil to develop three real leaves (3–4 weeks) in vegetation-favoring conditions (16:8 h photoperiod, 24 °C).

Plants with three fully developed leaves were artificially inoculated with *Xcc* by the infiltration of the inoculum through tiny openings created by a syringe needle. A needle-free syringe filled with the appropriate inoculum was applied directly on the leaf openings and 100 μL of the inoculum was infiltrated. Control plants were similarly treated with sterile deionized water. All plants were used in triplicates and were regularly inspected for symptoms until phenotypic symptoms visually appeared. Statistical analysis was performed using five leaves of control and an equal number of leaves from artificially infected plants to estimate the mean of optoacoustic signal progression for 24, 48 and 72 h after the infection, followed by the calculation of standard deviation. 

## 3. Results

**Figure 2:**
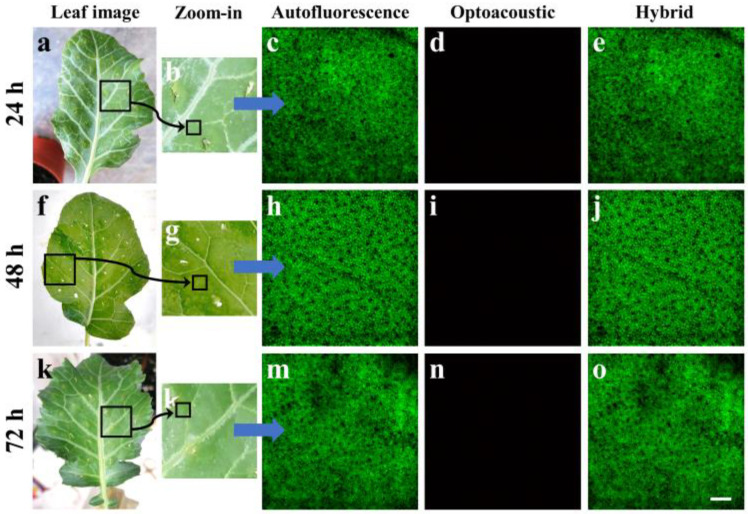
Hybrid imaging of control specimens. (**a**) Image of an extracted young broccoli leaf, 24 h after an injection with pure water. The black square represents the part of the leaf that was sectioned and placed in the sample holder of the microscope. (**b**) Zoom-in of the dissected region indicated with the black square in (**a**). The smaller black square shows the region that was finally scanned to obtain the hybrid images. (**c**) Autofluorescence image of a representative 1500 by 1500 μm^2^ area as indicated in (**b**). (**d**) Optoacoustic scan of the same region, showcasing a total absence of signals. (**e**) Hybrid image resulting through the merging of (**c**,**d**). Similar results recorded 48 (**f**–**j**) and 72 (**k**–**o**) hours post-injection. The scalebar is equal to 200 μm.

Aiming to demonstrate the capabilities of the hybrid system, we employed leaf samples from broccoli (*Brassica oleracea var. italica*) which had been artificially contaminated with *Xanthomonas campestris* pv. *campestris* (*Xcc*). *Xcc* is a well-known Gram-negative bacterium that causes black rot, a systemic vascular disease of brassica crops with no totally effective method of control. The leaves of the young broccoli plants were initially injected with pure water in several regions to act as control specimens for the realization of hybrid imaging measurements. Subsequently, the leaves were extracted from the plants at 24, 48 and 72 h post-injection and immediately imaged using the developed apparatus integrating confocal fluorescence and optoacoustic contrast modes. Figure 2a shows an image of a young broccoli leaf removed from the plant 24 h following the water injection. The black square, with an approximate area of 1 cm^2^, delineates the specific part of the leaf that was carefully excised and placed in the SH of the hybrid microscope (Figure 1) for the imaging session. Figure 2b shows a zoomed version of the previously selected leaf region, revealing various vessels with higher detail, while several injection holes are clearly visible in the periphery of the image. The smaller black square in the lower part of Figure 2b roughly indicates the representative area of 1500 by 1500 μm^2^ that was raster-scanned for hybrid data acquisition. In specific, the AF image (Figure 2c) demonstrates a rather homogeneous spatial distribution of signals which are predominantly emitted by chlorophyl pigment. Chlorophyl is present at high concentrations in plant cells and exhibits a high optical absorption coefficient for the utilized excitation wavelength at 450 nm as well as strong AF emission in the red part of the visible spectrum, overlapping with the detection range of the microscope (i.e., a 570 nm long-pass). It is worth mentioning that the spatial resolution of the system allows for the marginal discrimination of chloroplasts within the plant cells, appearing as dense round structures with a diameter in the order of 10 μm. In contrast with the recording of strong AF across the control leaf specimen, no appreciable optoacoustic signals were detected across the same region (Figure 2d). The apparent total absence of signals in the control specimen can be justified due to the negligible optical absorption and the strong reflection of the 532 nm (green) excitation wavelength by the chlorophyl pigment, which constitutes the primary chromophore in the plant cells. A merged image of the inspected region is additionally presented in Figure 2e, further highlighting the way in which AF detection prevails over OA detection. Similar images are presented for control specimens that were imaged 48 h (Figure 2f–j) and 72 h (Figure 2k–o) post-inoculation, demonstrating consistent results regarding the AF spatial distribution as well as the total absence of optoacoustic signals.

**Figure 3:**
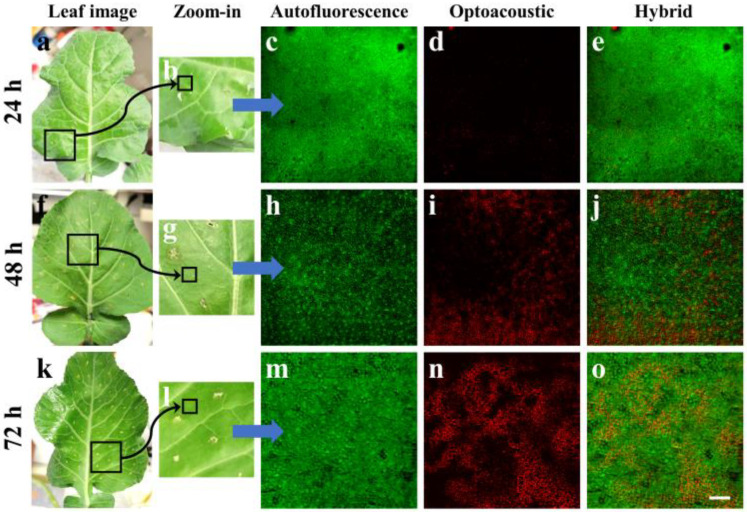
Hybrid imaging of *Xanthomonas campestris* pv. *campestris* (*Xcc*) artificially infected specimens. (**a**) Image of a detached young broccoli leaf, 24 h after an injection with *Xcc* bacteria. The black square represents the part of the leaf that was sectioned and placed in the sample holder of the microscope. (**b**) Zoom-in of the region indicated with the black square in (**a**). The smaller black square shows the region that was finally scanned to obtain the hybrid images. (**c**) Autofluorescence image of a representative 1500 by 1500 μm^2^ area as indicated in (**b**). (**d**) Optoacoustic scan of the same region, demonstrating low-amplitude sparsely distributed signals. (**e**) Hybrid image resulting through the merging of (**c**,**d**). Similar results recorded 48 (**f**–**j**) and 72 (**k**–**o**) hours post-injection, indicating a noteworthy increase of the optoacoustic signals. The scalebar is equal to 200 μm.

During the second part of the experiment, young broccoli leaves were artificially infected by injecting water containing a large concentration of *Xcc* bacteria directly into the vegetative tissue, in a similar manner to the previously presented control specimens. The leaves were again removed from the plant at 24, 48 and 72 h post-injection in order to be imaged by the hybrid microscopy system. Figure 3a shows an image of a young broccoli leaf 24 h after infection, with the black square indicating the part of the tissue that was removed and placed in the SH of the imaging apparatus. Figure 3b corresponds to the zoomed-in image of the sectioned tissue, whereas the small black square represents the area (1500 by 1500 μm^2^) that was raster-scanned by the hybrid microscope. It is worth mentioning that, despite the infection, these images of the leaf do not reveal any macroscopic signs of infection; on the contrary, they appear to be directly comparable to the control specimens presented in Figure 2.

An AF image of the selected region is shown in Figure 3c, demonstrating a homogeneous spatial distribution of signals mainly emitted by the chlorophyl pigment within the chloroplasts. Therefore, the AF image recorded at 24 h post-injection did not present any noteworthy alterations that could differentiate the infected from the control (i.e., healthy) specimens. On the other hand, Figure 3d shows the optoacoustic image for the same region, which in this case is characterized by low-amplitude, sparsely distributed signals covering the largest part of the examined tissue. Despite the fact that this image presents a detectable difference compared with the total optoacoustic signal absence observed in the control specimens (Figure 2), it may not yet be considered a reliable prognostic marker of the *Xcc* infection, as the emission is extremely low and could vary according to the investigated region of interest. A merging of the previous two images is presented in Figure 3e, highlighting the dominance of the emitted AF signals. However, 48 h after the *Xcc* injection, no macroscopic symptoms were visible in the cut broccoli leaves (Figure 3f–g). Nevertheless, while the respective AF image (Figure 3h) is similar to all previous cases (Figure 2), strong and spatially extended optoacoustic signals were detected as a result of the bacterial infection (Figure 3i). As has been demonstrated in preliminary measurements, *Xcc* bacteria in culture do not by themselves provide any optoacoustic signal, as they are virtually transparent at the excitation wavelength of 532 nm. Therefore, it can be safely deduced that the optoacoustic ultrasonic waves are emitted due to the significant alteration of the optical properties of the leaf tissue as a result of the infection. In contrast with the control specimens, which almost totally reflected the excitation light, the infected leaf appears to absorb an appreciable amount of optical radiation, an amount that is sufficient for providing detectable signals through the non-radiative relaxation of the molecules. A hybrid image merging the AF and optoacoustic maps is explicitly shown in Figure 3j to reveal a high contrast complementarity degree between the two microscopy modalities. Similar imaging data are presented in Figure 3k–o, as regards the imaging of a broccoli leaf at 72 h following the *Xcc* infection. In the case of the optoacoustic image (Figure 3n), we can observe apparently stronger and more extended signals than the respective inspection at 48 h (Figure 3i), covering the whole scanned region. This observation could be a direct indicator of the infection progress as *Xcc* bacteria continue to multiply, resulting in a gradually increasing alteration of the optical absorption properties in the leaf tissue. 

**Figure 4:**
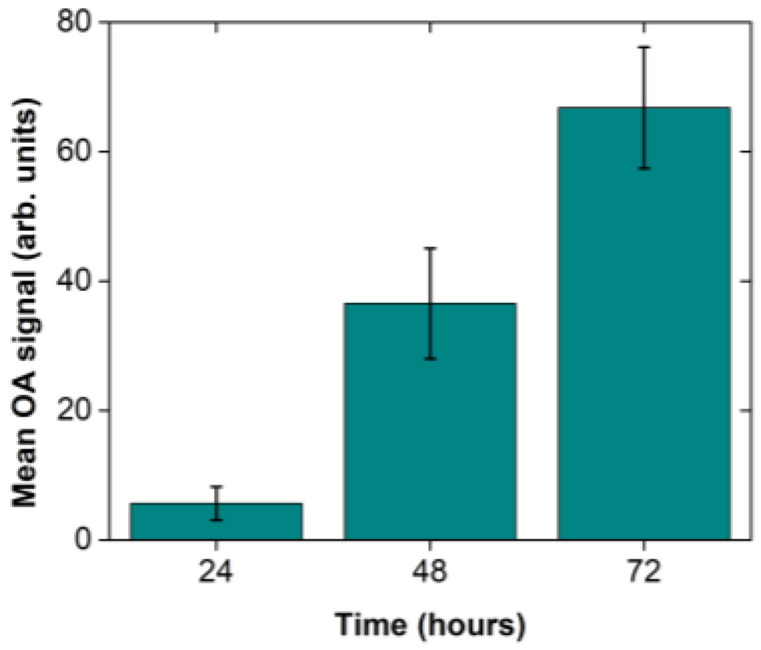
Mean optoacoustic (OA) signal progression for 24, 48 and 72 h after the infection. The mean values were estimated out of five optoacoustic images for each timepoint. Error bars represent the standard error of the mean.

Aiming at the quantification of the *Xcc* infection progress through the recording of optoacoustic signals, we have acquired five representative images for each of the selected timepoints at 24, 48 and 72 h after the infection using different broccoli leaves. The mean pixel value and the standard error of the mean were subsequently calculated out of the five measurements on the recorded images. The optoacoustic signal quantification (Figure 4) revealed a gradual increase of optoacoustic emission with time as the mean value (±1 standard deviation) increased from 5.66 ± 2.58 (24 h) to 36.54 ± 8.52 (48 h), and finally to 66.76 ± 9.36 at 72 h post the *Xcc* injection. Therefore, the extracted results indicate an approximately linear growth of the spatially averaged optical absorption coefficient of the tissue at 532 nm for the selected time range of observation.

## 4. Discussion 

The present study focuses on the application of innovative instrumentation to perform early and rapid identification of infected plants. The actual aim of this work was to demonstrate the capabilities of the developed hybrid microscopy system with regard to the possibility of an early detection of pathogenic bacterial infections in plant tissues prior to the onset of any macroscopic symptoms. The identification of infected plants was performed non-invasively and, most importantly, without the use of any chemical tags or specific fluorescent/optoacoustic contrast agents (label-free), requiring time-consuming preparation of the samples. This feature is crucial, as in plant produce the strategy is always to protect the consumer as well as the nurseryman or the scientist so that they are able to perform such analyses in the future. The development of such instrumentation is very important and needs to be as applicable as possible to imported nursery stocks as well as to perishable edible plant products to prevent disease spread and to prolong shelf or appropriate storage life, thus resulting in increased added value and economic benefit for the producer.

**Figure 5:**
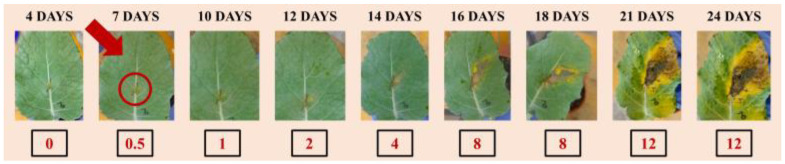
Development of disease symptoms caused by the pathogen *Xcc* in broccoli plant leaf (*Brassica oleraceae* var. *italica*) artificially infected during a 24 day period of observation. The numbers underneath each photo indicate the severity scale of the symptom (0: no symptom, 12: complete leaf damage due to typical black rot V-shaped lesion). The macroscopic detection of the disease symptom onset is indicated with a red arrow on the 7th day post-inoculation as the yellowing initiation inside the red circle.

The tested pathogen was the Gram-negative bacterium *Xanthomonas campestris* pv. campestris, the cause of black rot disease of crucifers and one of the most impactful bacterial plant pathogens based on economic importance for agriculture [27]. Symptoms in vegetable brassica crops, such as broccoli, normally appear during the 7th to 10th day [28]. In our work, macroscopic symptoms of the disease could be visually observed only after the 7th day of the artificial inoculation (Figure 5), as typical V-shaped yellow necrotic lesions (starting from the leaf margins) and blackening of the veins appear. In contrast, the hybrid imaging applied to highlight physiological changes that occur in plant tissues as a result of a pathogen-compatible interaction, enable us to detect a clear signal of infection immediately after 24 to 48 h. The attack of a plant by microbial pathogens activates plant defense responses and sets off a cascade of events to prevent or delay the infection symptoms. By combining the capabilities of confocal fluorescence and optoacoustic modalities, the proposed hybrid imaging methodology could be employed to detect physiological changes that are due to the presence of the pathogen.

More specifically, our target was the development of a rapid test able to detect the disease caused by *Xylella fastidiosa* (*Xf*) at early stages of infection, utilizing such innovative methodologies. As *Xf* is listed in the EPPO A2 list of quarantine pathogens and although new innovative plant protection agents have recently appeared [29,30,31], we could not work directly with it; thus, we opted to work with the well-studied pathosystem comprising broccoli plants and *Xcc*, a pathogen that shares common features with *Xf* on pathogenesis symptomatology effects. In the case of a proof of concept, we could extend its use to detect *Xf*, for example, at the entry points where propagating material is collected and inspected.

When broccoli leaves were challenged with the *Xcc*, optical absorption alterations were transcribed to plant cells, and the subsequent laser–tissue interaction led to the emission of optoacoustic signals that could be detected by our instrumentation (Figure 3). Although such signals may not have been *Xcc* specific, they could be correlated with the established infection as the control plant leaves, not challenged with the pathogen, did not present any similar reaction (Figure 2). More interestingly, the signal quantification process (Figure 4) showed that, only 24 h after the artificial inoculation, the instrument could reliably detect the tissue modifications induced by the pathogen. After 48 or 72 h the signal was further amplified, contrary to macroscopic symptoms that appear typically after the 7th to 10th day after the bacterial infection. Therefore, the proposed hybrid confocal fluorescence and optoacoustic microscopy imaging approach was positively utilized to detect the disease caused by a plant pathogen and can be further investigated for its ability to replace other classic or modern disease detection approaches.

## Data Availability

The content generated and analyzed in this study is available from the corresponding author on request.
